# [Corrigendum] Resveratrol improves neurological outcome and neuroinflammation following spinal cord injury through enhancing autophagy involving the AMPK/mTOR pathway

**DOI:** 10.3892/mmr.2026.13812

**Published:** 2026-01-28

**Authors:** Hong-Yu Meng, De-Cheng Shao, Han Li, Xiao-Dan Huang, Guang Yang, Bing Xu, Hai-Yun Niu

Mol Med Rep 18: 2237–2244, 2018; DOI: 10.3892/mmr.2018.9194

Subsequently to the publication of this paper, and following the publication of an expression of concern statement (doi: 10.3892/mmr.2025.13679) that was published after an interested reader had noted that, regarding the confocal microscopic images shown in Fig. 3 on p. 2240, the top (Sham) and bottom (SCI) data panels appeared to show a small overlapping section such that data which were intended to show the results from differently performed experiments had apparently been derived from the same original source, the authors have now replied to the Editorial Office. After re-examining their original data, the authors have realized that the data in Fig. 3 were inadvertently assembled incorrectly. The revised version of Fig. 3, now showing alternative data from one of the repeated experiments, is shown below. Note that this error did not significantly affect either the results or the conclusions reported in this paper, and all the authors agree with the publication of this corrigendum. Furthermore, the authors thank the Editor of *Molecular Medicine Reports* for granting them the opportunity to publish this corrigendum, and apologize to the readership for any inconvenience caused.

## Figures and Tables

**Figure 3.**
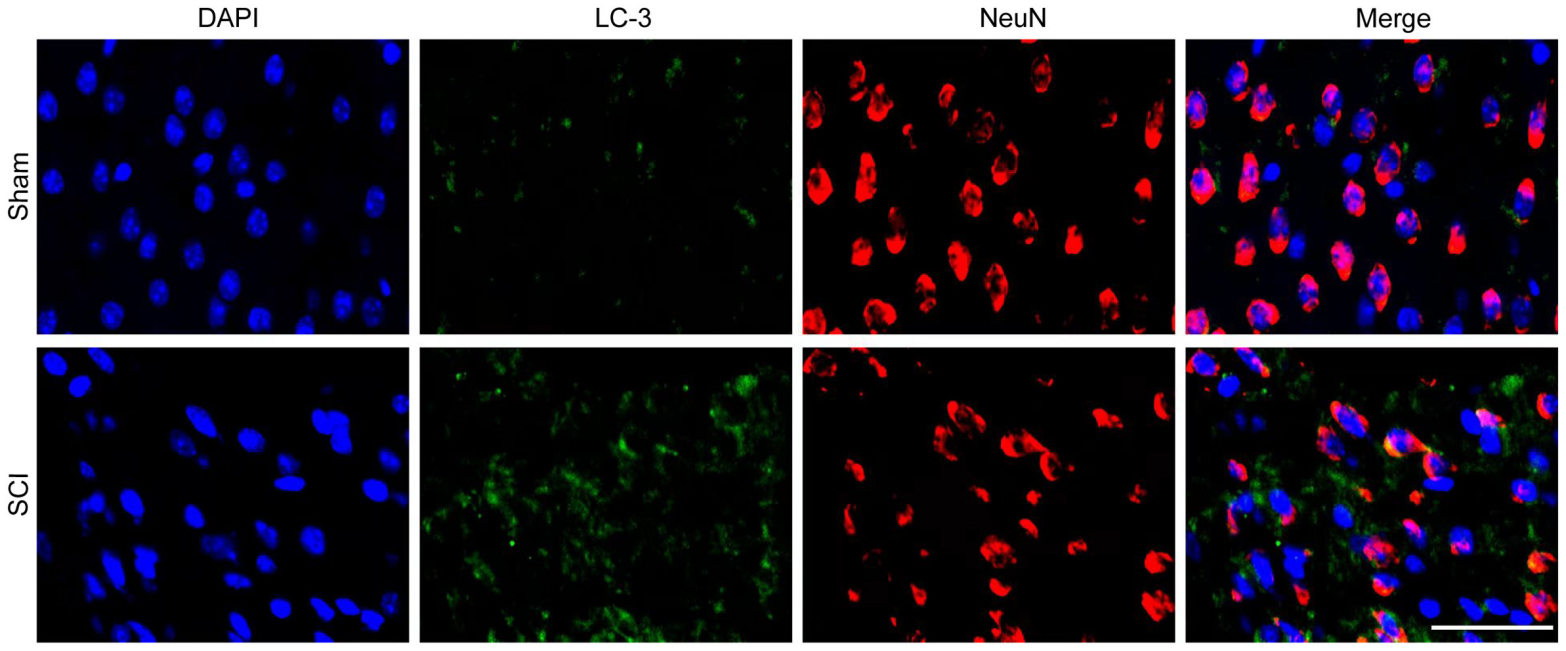
Confocal images of autophagic maker LC3 and NeuN. Immunofluorescence staining of LC3 and NeuN in the damaged spinal cord at 3 days post-SCI. The microphotographs were visualized by confocal laser scanning microscopy. Scale bar, 50 µm. SCI, spinal cord injury; LC3, microtubule-associated protein light chain 3; NeuN, neuronal nuclei.

